# Effects of Roasting Conditions on Antibacterial Properties of Vietnamese Turmeric (*Curcuma longa*) Rhizomes

**DOI:** 10.3390/molecules28217242

**Published:** 2023-10-24

**Authors:** Hai Thanh Nguyen, Siyuan Wu, Tomoki Ootawa, Hieu Chi Nguyen, Hong Thi Tran, Pitchaya Pothinuch, Hang Thi Thu Pham, Anh Thi Hong Do, Hao Thanh Hoang, Md. Zahorul Islam, Atsushi Miyamoto, Ha Thi Thanh Nguyen

**Affiliations:** 1 Department of Plant Biotechnology, Faculty of Biotechnology, Vietnam National University of Agriculture, Trau Quy Crossing, Gia Lam District, Hanoi 131000, Vietnam; nthaicnsh@vnua.edu.vn (H.T.N.); hang2904@gmail.com (H.T.T.P.); anhdo5511@gmail.com (A.T.H.D.); 2 Department of Veterinary Pharmacology, Joint Faculty of Veterinary Medicine Kagoshima University, 1-21-24 Korimoto, Kagoshima 890-0065, Japan; k0460053@kadai.jp (S.W.); k2773273@kadai.jp (T.O.); khokonpharma@gmail.com (M.Z.I.); 3 National Institute for Control of Vaccines and Biologicals, Hoang Mai District, Hanoi 128100, Vietnam; nguyenhieu210.nicvb@gmail.com (H.C.N.); tranngochong88@gmail.com (H.T.T.); 4 Faculty of Food Technology, Rangsit University, 52/347 Muang-Ake Pahonyontin Road, Lak-Hok, Pathum Thani 12000, Thailand; pitchaya.p@rsu.ac.th; 5 Department of Veterinary Pharmacology, Faculty of Veterinary Medicine, Vietnam National University of Agriculture, Trau Quy Crossing, Gia Lam District, Hanoi 131000, Vietnam; 650537@sv.vnua.edu.vn; 6 Department of Pharmacology, Faculty of Veterinary Science, Bangladesh Agricultural University, Mymensingh 2202, Bangladesh

**Keywords:** *Curcuma longa*, turmeric rhizome, antibacterial effect, roasting, curcumin

## Abstract

Processing with heat treatment has been reported to alter several therapeutic effects of turmeric. In Vietnamese traditional medicine, turmeric has been long used for bacterial infections, and roasting techniques are sometimes applied with this material. However, there have been no studies investigating the effects of these thermal processes on the plant’s antibacterial properties. Our study was therefore performed to examine the changes that roasting produced on this material. Slices of dried turmeric were further subjected to light-roasting (80 °C in 20 min) or dark-roasting (160 °C in 20 min) processes. Broth dilution and agar-well diffusion methods were applied to examine and compare the effects of ethanol extracts obtained from non-roasted, light-roasted and dark-roasted samples, on a set of 6 gram-positive and gram-negative bacteria. In both investigations, dark-roasted turmeric was significantly less antibacterial than non-roasted and light-roasted materials, as evident by the higher values of minimum inhibitory concentrations and the smaller diameters of induced inhibitory zones. In addition, dark-roasting was also found to clearly reduce curcumin contents, total polyphenol values and antioxidant activities of the extracts. These results suggest that non-roasting or light-roasting might be more suitable for the processing of turmeric materials that are aimed to be applied for bacterial infections.

## 1. Introduction

In Vietnamese traditional medicine, the thermal processing of turmeric (*Curcuma longa*) rhizomes as herbal materials might involve steaming before drying, or roasting after drying [1,2,3]. While steaming is considered as a suitable method that retains the natural color and aroma of turmeric, roasting, on the other hand, is believed to enhance the turmeric’s flavor and aroma, which could be preferable in recipes where a stronger turmeric flavor is desired [4]. In addition, it is likely that ethnopharmacological practitioners also select thermal processing methods based on the target medicinal uses of turmeric. According to Vietnamese pharmacopoeia [2], the roasting process is preferred for turmeric when materials are aimed to be applied within the circulatory system, such as for blood-tonifying or blood pressure regulation. With herbal plants used as medicine, roasting conditions have been reported to significantly affect the phytochemical contents [5], which therefore might alter their therapeutic effects [6]. In the case of turmeric, research has been focused on studying the effects of heat processing, including roasting, on the changes in phytochemical constituents [7], curcuminoid contents [8], antioxidant activities [9] and pharmacological functions such as anti-cancerogenic or anti-inflammatory effects [10,11]. However, it is likely that the influences induced by thermal processing on each therapeutic function of turmeric are different. For example, Sun et al. [9] observed that heat treatment decreased the neuroprotective functions of turmeric, while, in contrast, other studies reported that these processes enhanced the anti-cancerogenic and anti-inflammatory effects of curcumin [10,11]. The results therefore indicated that the influences of thermal processing nned to be verified in accordance with each target medicinal use. To the best of our knowledge, currently, there have been no studies investigating the effects of roasting on turmeric antibacterial properties. Therefore, the current study was performed to examine the changes that this thermal process induced on the effects of turmeric with bacteria. In addition, because the three main types of curcuminoids, including bisdemethoxycurcumin, demethoxycurcumin and curcumin, are usually considered as bioactive compounds responsible for turmeric’s medicinal uses [2,12], we also determined their contents, so as to verify if the changes in their concentrations are responsible for the alterations in antibacterial properties. In addition to that, polyphenol contents and antioxidant activities were measured, as these twophytochemical values have been reported to have relationships to the therapeutic uses of turmeric [13].

## 2. Results and Discussion

### 2.1. Effects of Roasting on Curcuminoid Contents of Turmeric

**Figure 1:**
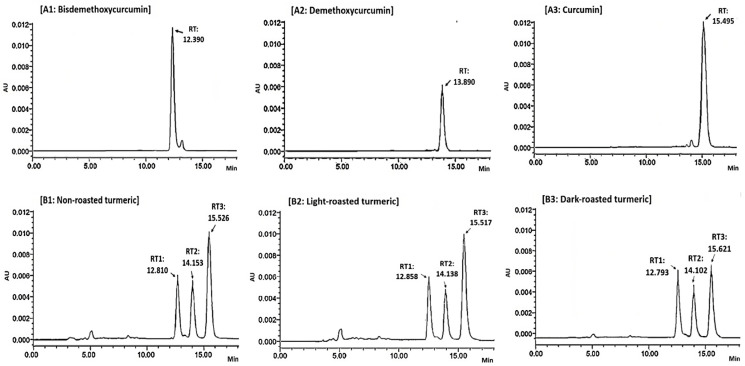
Representative HPLC chromatography of standards of (**A1**) bisdemethoxycurcumin (0.5 µg/mL), (**A2**) demethoxycurcumin (0.5 µg/mL), (**A3**) curcumin (1 µg/mL) and ethanol extracts (2 µg/mL) of (**B1**) non-roasted, (**B2**) light-roasted, (**B3**) dark-roasted turmeric samples.

Representative HPLC chromatograms of standard references and turmeric extracts are shown in Figure 1.

Retention time (RT) of bisdemethoxycurcumin, demethoxycurcumin and curcumin was 12.390 min (Figure 1(A1)), 13.890 min (Figure 1(A2)) and 15.495 min, respectively (Figure 1(A3)). In the chromatography of non-roasted turmeric (Figure 1(B1)), light-roasted turmeric (Figure 1(B2)) and dark-roasted turmeric (Figure 1(B3)) peaks with RT1 were identified as bisdemethoxycurcumin, while peaks with RT2 and RT3 were identified as demethoxycurcumin and curcumin, respectively.

The contents of each curcuminoid and total curcuminoids analyzed with non-roasted, light-roasted and dark-roasted turmeric samples are shown in Table 1.

Contents of curcumin, demethoxycurcumin, bisdemethoxycurcumin and total curcuminoid obtained from non-roasted turmeric used in our study were identified as 4.611 ± 0.042; 2.228 ± 0.110; 1.146 ± 0.071 and 7.985 ± 0.207 g, respectively, per 100 g of dried powder. These results were similar to previous research [14], which investigated turmeric samples in India and reported that the contents of total curcuminoids ranged from 5.95 to 13.05 g per 100 g of dried turmeric, and those of curcumin, demethoxycurcumin and bisdemethoxycurcumin ranged from 3.00 to 6.25 g, 1.62 to 3.50 g and 1.37 to 3.07 g, respectively, per 100 g dried turmeric. In addition, our results revealed that while light-roasting did not alter the contents of the three curcuminoids, dark-roasting process significantly reduced the contents of curcumin and total curcuminoids. However, all three samples, including the dark-roasted one, were still able to reach the standard requirements established in Vietnamese pharmacopoeia for turmeric materials applied as herbal medicine, which requires that the total curcuminoids are not less than 5% [2].

From our results, we observed that roasting with mild heat (light-roasting, 80 °C) did not alter the contents of all three types of curcuminoids. It was similar to previous studies, which also reported that curcumin, demethoxycurcumin and bisdemethoxycurcumin, both in pure and as mixture forms of curcuminoids, were thermally stable in conditions from 80 to 85 °C, because heating at this temperature for 2 h did not induce any changes in their concentrations [15,16]. However, roasting with high heat (dark-roasting) significantly decreased curcumin concentrations, from 4.611 ± 0.042 to 3.060 ± 0.030 g/100g of dried powder, which was also the main reason for the reduction in total curcuminoids, because contents of demethoxycurcumin and bisdemethoxycurcumin were not significantly altered by the process (Table 1). This can be explained by the fact that among the three curcuminoids, curcumin has been known to be the most susceptible in terms of various conditions, including thermal-induced degradation [11,16]. When converting to percentages, the loss of curcumin contents due to dark-roasting in our study was from 32.70 to 34.19%, which was similar to several previous studies which investigated the effects of high heat treatment on turmeric materials. Suresh et al. [8] reported the curcumin loss from boiling or pressure cooking of turmeric ranged from 27 to 53%. Similarly, Esatbeyoglu et al. [11] also observed that under model roasting conditions, curcumin is strongly degraded, because only 30% of the initial curcumin concentration was left after 5 min. When comparing with their results, the loss of curcumin due to high heat treatment in our study was less severe. This difference might be explained by the fact that the temperature established for roasting in our set of experiments was lower (160 °C vs. 180 °C). In addition, purified curcumin was applied in their set of experiments, while our study used turmeric powders, where other compounds such as demethoxycurcumin and bisdemethoxycurcumin were also co-existent. Previous research observed that the degradation extent of curcuminoids (in mixture form) was substantially less as compared to their pure form, therefore suggesting the synergistic stabilizing influence of demethoxycurcumin and bisdemethoxycurcumin on the curcumin contained in curcuminoid mixtures [16].

### 2.2. Effects of Roasting on Antibacterial Effects of Turmeric

#### 2.2.1. Effects of Roasting on MIC Values of Turmeric Ethanol Extracts

MIC values of turmeric ethanol extracts and curcumin are shown in Table 2.

In our study, MICs of ethanol turmeric extracts without roasting ranged from 62.5 to 250 µg/mL. In a previous study, Lawhavinit et al. [17] reported that MICs of ethanol turmeric extracts against bacteria ranged between 1.92 to 125 µg/mL. In addition, MIC of this extract against *S. aureus* 25923 was 31.25 µg/mL lower than our observation (125 µg/mL). This might be explained by the difference in turmeric samples and investigated methods, because their study applied agar dilution, and not broth dilution, in the detection of MIC values. Interestingly, we observed that the MIC of extracts exerted better antibacterial activities than purified curcumin against *B. subtilis*, as evidenced by the lower MIC value (62.5 vs. 125 µg/mL). These results were also similar to the report of Lawhavinit et al. [17], in which MICs of turmeric ethanol extracts were significantly lower than isolated curcuminoids against several bacteria, including *B. subtilis* (15.63 µg/mL vs. 125 µg/mL). Therefore, it is reasonable to suggest that compounds different from curcuminoids are significantly involved in the total effects of turmeric materials against bacteria, at least for the case of *B. subtilis*.

The MIC of curcumin against bacteria ranged from 125 to 250 µg/mL in our experiments. These results were similar to several previous studies, which reported that the MIC of curcumin against *B. subtilis* ATCC 6633 was 125 µg/mL [18] or 129 µg/mL [19]; the MIC against *P. aeruginosa* was 175 µg/mL; and MIC against *E.coli* was between 92.8 to 250 µg/mL [18,19,20], in which the MIC against *E.coli* ATCC 25922 was 163 µg/mL [19]; MIC against *S.* Typhimurium was 250 µg/mL [21]; and MIC against *S. aureus* was 187.5 to 500 µg/mL [19,20,22,23]. However, some results of our study were different from other observations. For examples, Mun et al. [22] reported that the MIC against *S. aureus* ATCC 25923 was 250 µg/mL, which is higher than results obtained with this study (125 µg/mL), and Zermeno-Ruiz et al. [24] reported that curcumin, up to a concentration of 330 µg/mL, exerted no inhibition on *Salmonella*. These differences might be explained by the different methods applied in MIC detection, and by the difference in the examined bacterial strains, because curcumin has been reported to exert very high selective antibacterial activities, as it could induce largely different effects even against bacteria of the same genus [25].

When comparing MICs obtained with the three turmeric samples, it is noticeable that MIC values of extracts from dark-roasted turmeric against all six tested bacteria were always higher than those from non-roasted or light-roasted samples (Table 2). The reduction in antibacterial effects of dark-roasted turmeric might be partly due to the decrement in curcumin content, as curcumin was also observed to exert inhibitory effects against all bacteria in our set of experiments (MIC ranged from 125 to 250 µg/mL) (Table 2).

#### 2.2.2. Effects of Roasting on Inhibitory Zones of Turmeric Ethanol Extracts

**Figure 2:**
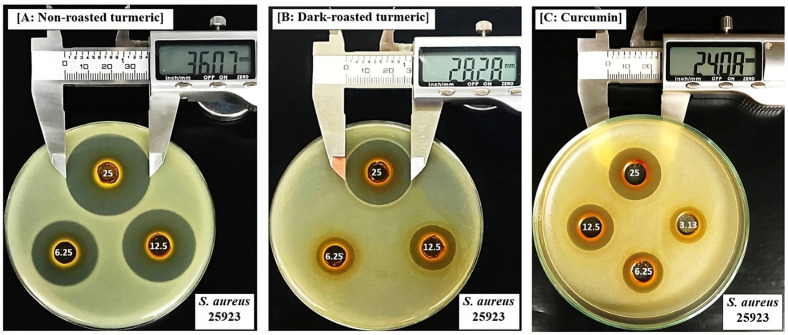
Inhibitory zone (mm) induced by ethanol extracts of (**A**) non-roasted turmeric, (**B**) dark-roasted turmeric and (**C**) curcumin on *Staphylococcus aureus* ATCC25923.

**Figure 3:**
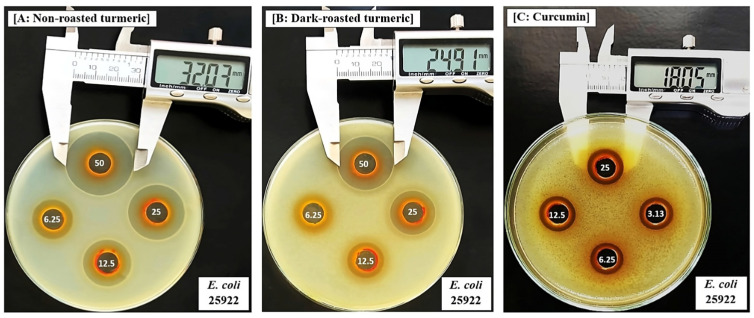
Inhibitory zone (mm) induced by ethanol extracts of (**A**) non-roasted turmeric, (**B**) dark-roasted turmeric and (**C**) curcumin on *Escherichia coli* ATCC25922.

Inhibitory zones (mm) induced by turmeric ethanol extracts and curcumin on bacteria are shown in Table 3 and Figure 2 and Figure 3.

When comparing the effects of the three turmeric samples, we observed that the dark-roasting process significantly reduced the antibacterial properties of the extracts, as evidenced by the significantly smaller inhibitory zones on both the gram (+) and gram (−) bacteria (Table 3). A previous study which investigated the effects of turmeric against bacteria also reported that ethanol extracts induced inhibitory zones on *S. aureus* ATCC25923, with the largest diameter of 16.55 ± 0.42 mm, which was smaller than our results (31.65 ± 1.09 mm) (Table 3 and Figure 2A). Furthermore, they observed no inhibition against *E. coli* ATCC25922, while our results showed that this extract induced clear inhibitory zones against this bacterium (Table 3 and Figure 3A). This difference can be explained by the difference in the origins of the turmeric, and by the different methods applied for investigation, as they used disc diffusion, and not agar-well diffusion, to determine inhibitory zones in their experiments.

In our study, curcumin exerted dose-dependent inhibitory zones on both gram bacteria, with the largest zone diameters obtained at 25 mg/mL and these ranged from 4.54 ± 0.56 (against *S.* Typhimurium) to 12.58 ± 1.39 (against *S. aureus*, Figure 2C). Similar to our study, Signh and Jain [26] also applied agar-well diffusion methods to investigate the effects of curcumin and observed that it could inhibit both of gram (+) and gram (−) bacteria, with the largest inhibitory zones varying from 8 to 20 mm. When normalizing the concentrations of extracts applied in each well of the agar diffusion method to contents of curcumin, the range of tested doses from 50 to 6.25 mg extract/mL were converted from 24.92 to 3.12 mg of curcumin/mL for non-roasted, 25.67 to 3.12 mg of curcumin/mL for light-roasted, and 16.54 to 2.07 mg of curcumin/mL for dark-roasted turmeric samples. Because the test with curcumin revealed that at these doses, curcumin was also able to exert significant inhibitory zones on the same set of experiments with the extracts (Table 3, Figure 2C and Figure 3C), we concluded that the decrement in curcumin contents, as a result of the dark-roasting process, is responsible, at least in part, for the reduction in antibacterial properties.

Interestingly, we observed that ethanol extracts could induced inhibitory zones that were larger than the maximum effects of curcumin at 25 mg/mL, against both gram (+) (Table 3 and Figure 2A vs. Figure 2C) and gram (−) bacteria (Table 2 and Figure 3A vs. Figure 3C). These results were similar to those obtained using the broth dilution method, in which the extract had a lower MIC value than curcumin against *B. subtilis* (62.5 vs. 125 µg/mL, Table 2). Among various compounds in turmeric, curcumin is the most well-known substance and therefore has obtained more attention and research interest. However, several studies have indicated that other groups, such as terpenoids, also play important roles in the therapeutic functions of *Curcuma* and related species [27,28]. For example, our previous study showed that turmeric induced relaxant effects on the cerebral artery in a manner that was dependent on its curcuminoid contents [29], and species of turmeric that contain no curcuminoids, such as *Curcuma amada* and *Curcuma zedoaria*, also exerted clear relaxation responses [29,30]. In other studies, curcumin–free turmeric showed anti-inflammatory and anticancer activities [31]. About its antibacterial properties, Wilson et al. [28] also reported that “white turmeric” without curcuminoid, such as *Curcuma zedoaria* and *Curcuma malabarica*, also induced high antibacterial activities, which were attributed to the presence of mono and sesquiterpenes. Non-curcuminoids from turmeric have also been reported to play important roles in other therapies, such as anti-inflammatory, antioxidant, anticancer [32] and anti-hypertensive [33] effects. A number of previous studies support the fact that in addition to curcumin, turmeric contains numerous other compounds that exhibit highly potent therapeutic functions [31]. In addition, curcuminoids that are different from curcumin, such as bisdemethoxycurcumin and demethoxycurcumin, have also been reported to act synergistically and potentiate the pharmacological functions of curcumin in anti-inflammatory [34,35], anti-proliferative [34] and nematocidal activities [36]. Results obtained with our current study, together with previous research, suggest that compounds different from curcumin are significantly involved in the antibacterial properties of turmeric. However, further research is still necessary to identify these compounds and elucidate their modes of action.

### 2.3. Effects of Roasting on Total Polyphenol and Antioxidant Activities of Turmeric

**Figure 4:**
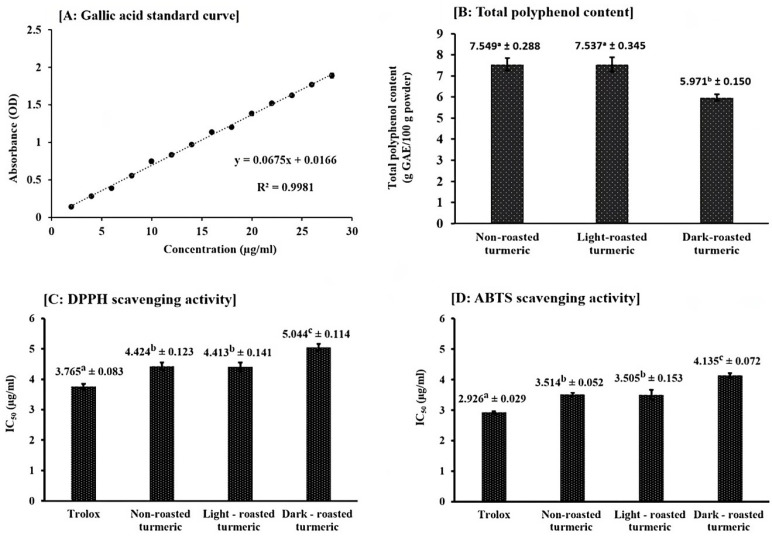
Results of total polyphenol contents and antioxidant activities of turmeric ethanol extracts from non-roasted, light-roasted and dark-roasted samples. (**A**): gallic acid standard curve, (**B**): total polyphenol content, (**C**): DPPH scavenging activity and (**D**): ABTS scavenging activity. Results are expressed as means ± standard deviation (SD) of three tests. Values with different superscript letters indicate significant difference (*p* < 0.05) compared with other values, assessed by one-way ANOVA followed with Tukey’s *post hoc* test.

Results on the measurement of total polyphenol contents are shown in Figure 4A,B. The total polyphenol content of non-roasted turmeric was determined to be 7.549 ± 0.288 g of gallic acid equivalent/100 g of dried turmeric samples. This result was similar to a previous study which also measured the total polyphenol contents of turmeric through ethanol extracts and reported that the values were between a range of 4.52 to 16.07 g of gallic acid equivalent/100 g of dried samples [37]. In addition, we observed that while light-roasting did not induce significant changes in total polyphenol contents, dark-roasting remarkably decreased these values (from 7.549 ± 0.288 to 5.971 ± 0.150 g of gallic acid equivalent/100 g of dried turmeric samples, as shown in Figure 4B).

Results on the IC_50_ values of turmeric samples, measured by DPPH and ABTS scavenging activities, are shown in Figure 4C,D. IC_50_ of trolox in DPPH and ABTS assays were identified as 3.765 ± 0.083 µg/mL and 2.926 ± 0.029 µg/mL, closely similar to the previous study of Wangsawat et al. [38], which identified those values at 0.004 mg/mL and 0.003 mg/mL, respectively. IC_50_ of ethanol extracts from non-roasting turmeric samples were identified at 4.424 ± 0.123 µg/mL in DPPH and 3.514 ± 0.052 µg/mL in ABTS scavenging activities. These results were lower than the previous study of Tanvir et al. [37], which reported that IC_50_ of this turmeric extract ranged from 1.08 to 3.03 µg/mL in DPPH. However, they were higher than those reported by Akter et al. [39] or Sabir et al. [40], who determined the values as 26.4 µg/mL or 27.2 µg/mL. This difference might be due to the difference in methods and the origins of the turmeric samples. Our study also revealed that dark-roasting significantly decreased the scavenging activities of turmeric extracts, as the IC_50_ values of dark-roasted turmeric were significantly higher than those of the non-roasted sample in both DPPH (5.044 ± 0.114 µg/mL vs. 4.424 ± 0.123 µg/mL, Figure 4C) and ABTS (4.315 ± 0.072 µg/mL vs. 3.514 ± 0.052 µg/mL, Figure 4D) assays.

Based on the results obtained with total polyphenol contents and free radical scavenging activities, we concluded that while light-roasting did not alter these values, dark-roasting significantly decreased them. In the previous study of Wu et al. [13], the stir-frying of dried turmeric rhizomes using mild heat was found to significantly potentiate both polyphenol contents and DPPH scavenging activities. However, these enhancements were not observed in our study, as the two values of the light-roasted samples remained the same as those of the non-roasted ones. This might be explained by the difference in heating conditions, because temperature and time established for mild heat stir-frying in their experiments were different from those of light-roasting in our current research. No previous research has investigated the effects of dark-roasting with high heat on the polyphenol and antioxidant activities of turmeric, and our results revealed that the process significantly decreased both of the two properties. The explanation for this reduction might be partly attributed to the decrement of curcumin contents in dark-roasted materials, as curcumin is one of the most important polyphenol compounds in turmeric and play an important role in its antioxidant activity.

Overall, our study observed that while light-roasting did not induce any negative effects on turmeric, dark-roasting significantly reduced its antibacterial properties, which can be partly explained via the decrement in curcumin contents. In addition, the process also decreased the values of polyphenol contents and antioxidant activities, which are known to be important in the various therapeutic effects of this herb. Therefore, we suggest that light-roasting is more preferrable for turmeric that is aimed to be used for bacterial infections. However, further roasting conditions should be tested in future studies to optimize the roasting process.

Turmeric has various traditional processing methods described in classical books of Materia medica, in which the conditions established for each thermal process are also different among countries, such as in China [13], India [10] and Vietnam [1,2]. It is likely that each processing method is established to meet the medicinal requirements for a specific clinical syndrome [13]. For example, Dahmke et al. [10] reported that heating curcumin with coconut fat or olive oil, similar to the traditional way that turmeric was cooked in India, could increase its anti-cancerogenic properties, because compounds derived from the pyrolysis of curcumin due to heating treatment with these particular fats could exert higher therapeutic effects against tumor cells than the initial curcumin. Wu et al. [13], when examining five methods of traditional stir-frying conditions established for turmeric, including stir-frying and stir-frying with wine, vinegar, water extract of rice and mussel powder processing, reported that different processing technologies had influenced the quality and quantity of curcuminoids to different degrees. Even though the relationships between changes in the curcuminoid profiles of the five different processed turmeric samples with their pharmacological activities have not yet established, these results provide scientific evidence explaining why each medicinal use requires specified methods of processing [13]. Interestingly, in addition to the traditional methods described in the classical books of Materia Medica, modern processing techniques, such as puffing, were also reported to significantly enhance the antioxidant and anti-inflammatory properties of turmeric [41,42]. Therefore, we suggest that roasting with more specified conditions should be further examined in future studies, in order to find out the optimal conditions to process turmeric materials with antibacterial uses. In addition, such information is also necessary to provide explanations and give an insight into various traditional processing methods.

## 3. Materials and Methods

### 3.1. Plant Material and Extraction

Turmeric rhizomes were prepared following our previous study [29], with some modifications. Fresh rhizomes were supplied by Vuon Duoc Lieu Herbarium, Vietnam National University of Agriculture (Hanoi, Vietnam). The plant identity was confirmed by Dr Tho Thi Bui, based on the voucher specimens that have been deposited at the Vietnam National University of Agriculture. The provider certified that products reached the Vietnam National Standards for herbal materials used as medicine, established in Vietnamese Pharmacopoeia by Vietnam Ministry of Health, which requires that the content of curcuminoids in dried turmeric rhizomes is not less than 5%. The rhizomes were then washed, cut into 2 mm slices with a fresh-rhizome slicer (Model: TMTP-O22, Tan Minh instruments, Hanoi, Vietnam) before being oven-dried at 60 °C for 96 h to obtain a constant weight. The roasting process was performed following the “General Guidance on the Traditional Processing of medicinal plants” (Vietnam Ministry of Health, Circular Number 30/2017/TT-BYT, issued 2017) [43], applying the herbal roasting machine (Model: TMND-B06, Tan Minh instruments, Hanoi, Vietnam). The conditions for mild heat roasting (light-roasting) were 80 °C for 20 min, while those for high heat (dark-roasting) were 160 °C for 20 min, with the speed of the drum rotation selected at 30 rpm. Slices of non-roasted, light-roasted and dark-roasted turmeric were then ground into powder with a coffee blender before passing through a sieve with a nominal mesh size of 2 mm. Extractions were performed following our previous study [44], with some modifications. Ethanol was selected as the extracting solvent because it has been identified as the most preferred solvent for the antibacterial effects of turmeric [17]. Briefly, 10 g of powder was stirred with 300 mL of ethanol and left at room temperature for 1 day of absorbance. The mixtures were then filtered through two layers of cheese cloth, centrifuged at 10,000× *g* for 30 min and finally were passed through grade No. 2 qualitative filter paper (Advantec MFS Inc., Dublin, CA, USA) to remove all precipitations. Filtrates were then concentrated at 37 °C using a rotary evaporator at low atmospheric pressure to remove all solvents and we obtained the dried extracts. These final dry weights were then used to calculate extraction yield (%), which was determined at 9.25% for all of non-roasted, light-roasted and dark-roasted turmeric samples applied in this study. All extracts were kept in a refrigerator at 4 °C for experimental analyses.

### 3.2. Reagents and Bacterial Strains

*Bis*demethoxycurcumin, demethoxycurcumin and curcumin references at analytical standards (purity of ≥98%) and gallic acid (≥97%) were purchased from Sigma-Aldrich (St. Louis, MO, USA). Next, 1,1-Diphenyl-2-picrylhydrazyl (DPPH) and 2,2′-Azino-bis (3-ethylbenzo thiazoline-6-sulfonic acid) (ABTS) were purchased from WAKO Pure Chemical (Osaka, Japan). Trolox was purchased from Calbiochem (San Diego, CA, USA). Ethanol, HPLC grade acetonitrile, HPLC grade acid acetic and other compounds or solvents, at analytical levels, were purchased from Merck (Darmstadt, Germany). Tested bacteria, including two gram-positive (gram (+)), such as *Bacillus subtilis* (*B. subtilis*) ATCC 6633 and *Staphylococcus aureus* (*S. aureus*) ATCC 25923, as well as four gram-negative (gram (−)), such as *Escherichia coli* (*E. coli*) ATCC 25922, *E. coli* ATCC 85922, *Pseudomonas aeruginosa* (*P. aeruginosa*) ATCC 9027 and *Salmonella enterica* subsp. *enterica* serovar Typhimurium (*S.* Typhimurium) ATCC 13311, were purchased from the American Type Culture Collection (Rockville, MD, USA).

### 3.3. HPLC Analysis of Curcuminoids

Analysis of curcumin, bisdemethoxycurcumin and demethoxycurcumin was performed using HPLC techniques, following the “General instructions for the determination of curcuminoid content by HPLC method”, which have been established by the National Institute for Food control and accredited by the Vietnam Standards and Quality Institute (Code NIFC.05.M.132. Documentary number: 894.2020/QĐ-VPCNCL, issued 2020) [45]. Briefly, a system consisting of an Agilent C18 (250 mm × 4.6 mm × 5 µm) column, which was connected to a 1260 Agilent HPLC (Agilent Technologies, Palo Alto, CA, USA) and equipped with a PDA detector, was used throughout the analysis. The mobile phase comprised a 1% acid acetic: acetonitrile (55:45 *v*/*v*). The flow rate was set at 1 mL/min and the injection volume was 20 µL. Curcuminoids were detected at 420 nm. The urcumin, bisdemethoxycurcumin and demethoxycurcumin contents in the samples were calculated by comparing the sample peak areas (% fluorescence) with the standard curve of these purified standards. Analysis was performed in triplicate.

### 3.4. Evaluation of Antibacterial Effects of the Extracts

The effects of extracts on bacteria were evaluated through broth dilution and agar-well diffusion methods. In order to observe the dose-dependent effects, dimethyl sulfoxide (DMSO) was applied to the diluted extracts and purified curcumin to obtain serial tested concentrations.

The broth dilution method was performed to determine minimum inhibitory concentration (MIC) values, following the methods of the Clinical and Laboratory Standards Institute [46], and with some modifications to adjust the conditions for testing the plant materials [47]. Tested solutions were mixed with Muller–Hinton broth in 96-well microplates to produce serial dilutions ranging from 500 µg/mL to 3.91 µg/mL. Final bacterial concentration was adjusted at 5 × 10^5^ cfu/mL. All bacteria were incubated at 37 °C for 24 h. The lowest concentration displaying no visible growth was recorded as the MIC. DMSO served as a negative control and kanamycin was applied as a positive and quality control, with MIC against *E. coli* ATCC 25922 determined at 2 µg/mL, which was within the acceptable limits (from 1–4 µg/mL) established by the Clinical and Laboratory Standards Institute [46].

The gar-well diffusion method was performed following Gonelimali et al. [48], and with some modifications. Briefly, a Muller–Hinton agar plate was inoculated with bacteria at a final concentration of 10^6^ cfu/mL, and a hole with a diameter of 10 mm was punched aseptically with a cork borer. A total of 100 µL of tested materials, including extracts and purified curcumin at established concentrations, was added into the well. Agar plates were incubated under 37 *°*C for 24 h and inhibitory zones (excluding 10 mm of well diameter) were measured. DMSO induced no inhibition and was applied as a negative control. Experiments were performed in triplicate.

### 3.5. Determination of Total Polyphenol and Antioxidant Activities

Evaluation of total polyphenol contents: total polyphenol contents were measured following the method of Suda et al. [49], with some modifications. For control, to 0.2 mL of extracts diluted with DMSO was added 1 mL of Folin ciocalteu. This mixture was allowed to stand at room temperature for 3 min. Then, 1 mL of 10% Na_2_CO_3_ and 5 mL of distilled water were added. After one hour of incubation, the absorbance was measured using a spectrophotometer (ERMA model AE-450, Saitama, Japan) at 750 nm. For the blank, a sample without Folin ciocalteu reagent was taken along as well. All experiments were performed in triplicate, and gallic acid was used as a standard substance. The total polyphenol contents were expressed as g gallic acid equivalent per 100 g of dry samples.

Evaluation of antioxidant activities: the antioxidant activities of the extracts were evaluated through DPPH and ABTS radical scavenging activity.

DPPH scavenging activity was measured with DPPH, according to the procedure described by Masuda et al. [50], and with some modifications. DPPH solution was made by mixing 0.1 g of DPPH powder with 50 mL of ethanol at 96%. Briefly, to 0.2 mL of extracts diluted with DMSO was added 0.1 mL of DPPH solution and 4.8 mL of DMSO. The absorbance was determined using the spectrophotometer at a 515 nm wavelength with a blank containing only the sample and solvent. For the control absorbance measurement, to a 0.1 mL DPPH solution was added 4.9 mL DMSO solvent. The result was expressed as a percentage of the antioxidant activity, which was calculated using the equation:

DPPH radical scavenging (%) = [Ac − (As − Ab)/Ac] × 100

where Ac (A control) is the absorbances of DMSO and DPPH solution; As (A sample) is the absorbance of extracts and DPPH solution; and Ab (A blank) is the absorbance of extracts and DMSO.

ABTS scavenging activity was measured with ABTS, according to the procedure described by Wetwitayaklung et al. [51], and with some modifications. To produce an ABTS^+^ radical solution, 7 mM of ABTS in water was reacted equally with 4.9 mM of potassium persulfate (K_2_S_2_O_8_) in water. This mixture was allowed to stand in the dark at room temperature for 12 to 16 h. The working solution was prepared by diluting ABTS^+^ radical solution with water until the absorbance was 0.70 ± 0.02 at 734 nm using a spectrophotometer. In the assay, 0.2 mL of extracts diluted with DMSO was mixed with 3.8 mL of ABTS^+^ radical solution. The absorbance at 734 nm was determined after 4 min. For each sample, a blank was prepared with 3.8 mL of water, instead of the ABTS^+^ radical solution. Trolox was used as a positive control. All samples were evaluated in triplicate. The % ABTS radical scavenging activity was calculated using the equation:

ABTS radical scavenging (%) = [Ac − (As − Ab)/Ac] × 100

where Ac (A control) is the absorbances of DMSO and ABTS solution; As (A sample) is the absorbance of extracts and ABTS solution; and Ab (A blank) is the absorbance of extracts and DMSO.

All experiments in the measurements of DPPH and ABTS scavenging activities were performed in triplicate and trolox was used as a standard substance. The plotted graphs between % scavenging and concentration were used to calculate the half maximal inhibitory concentration (IC_50_) values, the concentrations that are required to inhibit 50% of DPPH or ABTS radical scavenging.

### 3.6. Statistical Analysis

Results are expressed as means ± standard deviation (SD). Statistical analyses were performed by unpaired *t*-test or the Tukey’s test after one-way analysis of variance (one-way ANOVA). Significance was established when the probability level was equal to or less than 5%.

## 4. Conclusions

Our study, for the first time, has investigated the effects of the roasting process on the antibacterial functions of turmeric. While roasting with mild heat (light-roasting) did not alter the effects of turmeric on bacteria, we observed that roasting with high heat (dark-roasting) significantly decreased antibacterial properties, as well as the curcumin contents and other therapeutic values, such as total polyphenol and antioxidant activities. We therefore suggest that light-roasting might be more preferred in the processing of turmeric for bacterial infections. However, further research is necessary to elucidate the relationships between changes in the phytochemical profiles and the alteration in the antibacterial effects in processed materials.

## 5. Limitations

Our study has several limitations that should be verified in future research. Firstly, we have not yet identified the products derived from thermal degradation of curcumin to clarify how the changes in phytochemical constituents participate in the reduction in antibacterial properties. In addition, research on animals is needed to confirm the comparison results with in vivo conditions. Furthermore, investigations with more specified processing conditions should be tested to find out the optimal roasting methods for turmeric.

## Figures and Tables

**Table 1:** Contents of curcumin, bisdemethoxycurcumin, demethoxycurcumin and total curcuminoids (g/100 g dried powder) in non-roasted turmeric, light-roasted turmeric and dark-roasted turmeric samples.

Material	Curcumin	Demethoxy-Curcumin	Bisdemethoxy-Curcumin	Total Curcuminoid
Non-roasted turmeric	4.611 ^a^ ± 0.042	2.228 ± 0.110	1.146 ± 0.071	7.985 ^a^ ± 0.207
Light-roasted turmeric	4.635 ^a^ ± 0.073	2.157 ± 0.127	1.154 ± 0.043	7.945 ^a^ ± 0.064
Dark-roasted turmeric	3.060 ^b^ ± 0.030	2.168 ± 0.225	1.149 ± 0.028	6.376 ^b^ ± 0.197

Results are expressed as means ± standard deviation (SD) of three tests. Values with different superscript letters indicate significant difference (*p* < 0.05) compared with other values of the same column, assessed by one-way ANOVA followed with Tukey’s *post-hoc* test.

**Table 2:** Minimum inhibitory concentration (µg/mL) of ethanol extracts of turmeric and curcumin.

Tested Material	Gram (+)		Gram (−)			
	*B. subtilis*	*S. aureus*	*E. coli* 25922	*E. coli* 85922	*S.* Typhimurium	*P. aeruginosa*
Non-roasted turmeric	62.5	125	250	250	250	250
Light-roasted turmeric	62.5	125	250	250	250	250
Dark-roasted turmeric	125	250	500	500	500	500
Curcumin	125	125	250	250	250	250

*B. subtilis*, *Bacillus subtilis* ATCC 6633; *E. coli*, *Escherichia coli*; *S. aureus*, *Staphylococcus aureus* ATCC 25923; *S.* Typhimurium, *Salmonella* Typhimurium ATCC 13311; *P. aeruginosa*, *Pseudomonas aeruginosa* ATCC 9027.

**Table 3:** Inhibitory zones (mm) induced by turmeric ethanol extracts and curcumin on bacteria.

Bacterium		Material	Concentration (mg/mL)			
Gram (+)	*B. subtilis*	Extract	50 mg/mL	25 mg/mL	12.5 mg/mL	6.25 mg/mL
		Non–roasted	24.25 ^a^* ± 0.14	21.29 ^a^ ± 0.57	17.73 ^a^ ± 1.10	8.41 ^a^ ± 0.75
		Light–roasted	24.18 ^a^ ± 0.73	20.91 ^a^ ± 0.76	17.66 ^a^ ± 0.79	8.44 ^a^ ± 1.28
		Dark–roasted	14.91 ^b^ ± 1.33	14.23 ^b^ ± 1.06	10.89 ^b^ ± 0.67	3.44 ^b^ ± 0.46
		Curcumin	25 mg/mL	12.5 mg/mL	6.25 mg/mL	3.13 mg/mL
			10.42 ± 0.70	7.80 ± 0.23	5.04 ± 0.64	3.07 ± 0.26
	*S. aureus*	Extract	50 mg/mL	25 mg/mL	12.5 mg/mL	6.25 mg/mL
		Non–roasted	31.65 ^a^* ± 1.09	25.89 ^a^ ± 0.16	14.85 ^a^ ± 0.72	10.06 ^a^ ± 0.90
		Light–roasted	31.19 ^a^ ± 0.43	25.40 ^a^ ± 0.69	15.21 ^a^ ± 1.97	9.91 ^a^ ± 1.06
		Dark–roasted	20.91 ^b^ ± 0.85	17.57 ^b^ ± 0.67	7.22 ^b^ ± 0.65	3.71 ^b^ ± 0.28
		Curcumin	25 mg/mL	12.5 mg/mL	6.25 mg/mL	3.13 mg/mL
			12.58 ± 1.39	9.64 ± 0.76	6.61 ± 1.09	3.06 ± 0.06
Gram (−)	*E. coli* ATCC 25922	Extract	50 mg/mL	25 mg/mL	12.5 mg/mL	6.25 mg/mL
		Non–roasted	20.36 ^a^* ± 1.63	10.79 ^a^ ± 0.33	7.95 ^a^ ± 0.39	5.00 ± 0.91
		Light–roasted	20.55 ^a^ ± 1.23	10.12 ^a^ ± 1.29	7.78 ^a^ ± 0.86	5.03 ± 1.16
		Dark–roasted	14.34 ^b^ ± 0.69	7.39 ^b^ ± 0.85	4.70 ^b^ ± 0.47	-
		Curcumin	25 mg/mL	12.5 mg/mL	6.25 mg/mL	3.13 mg/mL
			7.99 ± 0.12	5.66 ± 0.78	3.74 ± 1.03	-
	*E. coli* ATCC 85922	Extract	50 mg/mL	25 mg/mL	12.5 mg/mL	6.25 mg/mL
		Non–roasted	16.78 ^a^* ± 2.26	12.56 ^a^ ± 2.05	9.66 ^a^ ± 1.17	5.57 ± 0.79
		Light–roasted	17.11 ^a^ ± 0.47	12.33 ^a^ ± 3.82	8.99 ^a^ ± 0.95	5.66 ± 1.84
		Dark–roasted	11.68 ^b^ ± 0.33	7.66 ^b^ ± 0.84	5.25 ^b^ ± 0.58	-
		Curcumin	25 mg/mL	12.5 mg/mL	6.25 mg/mL	3.13 mg/mL
			7.81 ± 0.24	5.78 ± 0.58	3.45 ± 0.71	-
	*P. aeruginosa*	Extract	50 mg/mL	25 mg/mL	12.5 mg/mL	6.25 mg/mL
		Non–roasted	12.51 ^a^* ± 0.83	9.66 ^a^ ± 0.69	7.02 ^a^ ± 0.32	3.63 ± 0.13
		Light–roasted	11.64 ^a^ ± 0.85	9.66 ^a^ ± 1.74	7.01 ^a^ ± 0.69	3.75 ± 0.52
		Dark–roasted	9.79 ^b^ ± 0.34	7.46 ^b^ ± 0.47	4.05 ^b^ ± 0.27	-
		Curcumin	25 mg/mL	12.5 mg/mL	6.25 mg/mL	3.13 mg/mL
			7.77 ± 0.50	3.76 ± 0.52	-	-
	*S.* Typhimurium	Extract	50 mg/mL	25 mg/mL	12.5 mg/mL	6.25 mg/mL
		Non–roasted	9.85 ^a^* ± 0.42	7.30 ^a^ ± 0.52	6.27 ± 0.31	5.40 ± 0.45
		Light–roasted	10.12 ^a^ ± 1.18	7.77 ^a^ ± 0.67	6.44 ± 0.68	5.17 ± 0.81
		Dark–roasted	6.15 ^b^ ± 0.86	4.74 ^b^ ± 1.20	-	-
		Curcumin	25 mg/mL	12.5 mg/mL	6.25 mg/mL	3.13 mg/mL
			4.54 ± 0.56	3.07 ± 0.13	-	-

Results are expressed as means ± standard deviation (SD) of three tests. - means no inhibition. Values with different superscript letters indicate significant difference (*p* < 0.05) compared with other values from different extracts or compounds at similar concentrations on the same bacterium, assessed by one-way ANOVA followed with Tukey’s *post hoc* test. Values with superscripted * and bold letters indicate significant difference (*p* < 0.05) between the maximum inhibition zone induced by non-roasted turmeric vs. that of the curcumin, assessed by unpaired *t*-test. *B. subtilis*, *Bacillus subtilis* ATCC 6633; *E. coli*, *Escherichia coli*; *S. aureus*, *Staphylococcus aureus* ATCC 25923; *S.* Typhimurium, *Salmonella* Typhimurium ATCC 13311; *P. aeruginosa*, *Pseudomonas aeruginosa* ATCC 9027.

## Data Availability

All the data are shown in manuscript and Supplementary Materials (S1 Data).

## Supplementary Materials

The following supporting information can be downloaded at: https://www.mdpi.com/article/10.3390/molecules28217242/s1, Supplementary materials: S1 Data (ZIP).

## Abbreviations

ABTS: 2,2′-Azino-bis (3-ethylbenzo thiazoline-6-sulfonic acid; DPPH: 1,1-Diphenyl-2-picrylhydrazyl; DMSO: dimethyl sulfoxide; *Gram-positive: gram* (+); *Gram-negative: gram* (−); MIC: minimum inhibitory concentration; IC50: half maximal inhibitory concentration; RT: retention time; *B. subtilis: Bacillus subtilis; E. coli: Escherichia coli; P. aeruginosa: Pseudomonas aeruginosa; S. aureus: Staphylococcus aureus; S.* Typhimurium: *Salmonella enterica* subsp. *enterica* serovar Typhimurium.
